# High serum FSH levels on day 7 of stimulation are negatively associated with oocyte retrieval in GnRH antagonist protocols: real-world evidence from 9,969 IVF cycles in China

**DOI:** 10.3389/fendo.2026.1757107

**Published:** 2026-05-05

**Authors:** Xiu Luo, Hong Ye, Li Pei, Yao He, Fujie Li, Wei Han, Shun Xiong, Shubiao Han, Jingyu Li, Xiaodong Zhang, Guoning Huang

**Affiliations:** 1 Women and Children’s Hospital of Chongqing Medical University, Chongqing, China; 2 Chongqing Health Center for Women and Children, Chongqing, China; 3 Key Laboratory of Human Embryo Engineering, Chongqing Maternal and Child Health Care Hospital, Chongqing, China

**Keywords:** GnRH antagonist, *in vitro* fertilization, oocyte retrieval, serum FSH, threshold

## Abstract

This study aimed to investigate how levels of serum follicle-stimulating hormone (FSH) on Day 7 of ovarian stimulation are related to the number of oocytes retrieved, and identify any cutoff or threshold point in protocols using gonadotropin-releasing hormone (GnRH) antagonists. We examined data from January 2017 to June 2024, covering 9,969 cycles from 7,981 patients using GnRH antagonists as part of *in vitro* fertilization processes. We found a complex link between serum FSH and oocyte collection. Lower serum FSH on Day 7 was associated with collection of fewer oocytes (β = −0.531, p < 0.0001), and different levels were associated with changes in the number of oocytes collected. The key point was 9.13mIU/mL. If serum FSH exceeded 9.13 mIU/mL on Day 7, each 1mIU/mL increase reduced the number of oocytes collected by 0.07 (effect β = −0.07, 95% confidence interval [CI] −0.10 to −0.05, P < 0.0001). When serum FSH was below 9.13 mIU/mL, each 1 mIU/mL rise increased the number of oocytes collected by 1.18 (effect β = 1.18, 95% CI 0.95 to 1.41, P < 0.0001). This association suggests that it may be possible to improve ovarian reaction by raising the FSH dose if serum FSH is under 9.13 mIU/mL. However, if serum FSH is already over 10 mIU/mL, it seems likely that other steps may be needed to increase ovarian reaction, although these hypotheses will need to be tested in future studies.

## 1 Introduction

*In vitro* fertilization (IVF) involves ovarian stimulation using follicle-stimulating hormone (FSH) to enhance oocyte production. However, despite improvements in assisted reproductive technology in recent years, the best FSH dosage strategy is still not fully clear, and understanding how serum FSH levels are associated with oocyte collection is important for better IVF outcomes.

Some recent studies have found that giving more FSH results in more oocytes collected and higher serum FSH levels (1–3). High serum FSH seems to be associated with a greater ovarian reaction. However, other studies have found that high serum FSH is not always associated with the collection of more oocytes (4, 5). It has been speculated that excessive serum FSH might harm oocyte quality and how well the womb accepts an embryo, possibly leading to poorer IVF results. For example, one study found that if FSH levels are over 22IU/L by Day 7 of stimulation, adding more FSH might not grow more follicles (5). Higher FSH might lead to more oocytes initially (6), but can harm the quality of oocytes and embryos, potentially lowering birth rates (7). This suggests the need for custom treatments that focus on both the quality and quantity of ovarian stimulation.

Researchers have studied FSH levels and ovarian stimulation extensively, but we still do not know the exact FSH levels needed to best balance quality and quantity of oocytes. Results are conflicting, with some studies even reporting no connection between serum FSH levels and the number of oocytes retrieved after a set FSH dose (8). Factors influencing serum FSH levels during ovarian stimulation include the types (urinary or recombinant FSH), dose and route of administration (intravenous or subcutaneous) of exogenous FSH (4, 8, 9), patient body weight, body mass index (BMI), and the volume of drug distribution (8). FSH derived from distinct sources (urinary or recombinant) has different glycosylation patterns, leading to heterogeneous pharmacokinetics, pharmacodynamics, receptor-binding activity, and systemic clearance, all of which can influence serum concentration (10). We also know that serum FSH concentrations reach a steady state after 5–7 days of continuous rFSH administration (11). Studies have found that during controlled ovarian stimulation, the FSH concentration in follicular fluid is significantly influenced by and nearly equal to that in serum (12). FSH stimulates follicular growth and development by binding to receptors on granulosa cells of the follicles, while simultaneously being cleared from the blood circulation (13–15). Ovarian responsiveness to gonadotropins such as FSH largely depends on the level of expression of FSH receptors in the granulosa cells. The steady-state serum FSH level achieved after 5–7 days of ovarian stimulation reflects the balance between FSH absorption and clearance rates (16). Serum FSH level can therefore be used to assess the appropriateness of the FSH dose, and previous studies have selected Day 7 of ovarian stimulation as an indicator of stimulation intensity (5). We therefore used the same timing to support consistency and comparability across studies.

This retrospective cohort study examined IVF cycles taking place from January 2017 to June 2024 at Women and Children’s Hospital of Chongqing Medical University. The aim was to understand how Day 7 serum FSH levels are related to the number of oocytes retrieved and thus better understand ovarian response. This in turn would generate low-cost and dependable findings that could provide hypotheses for future research that might then guide future clinical approaches to increase the chances of success in IVF.

## 2 Materials and methods

### 2.1 Study design and population

**Figure 1:**
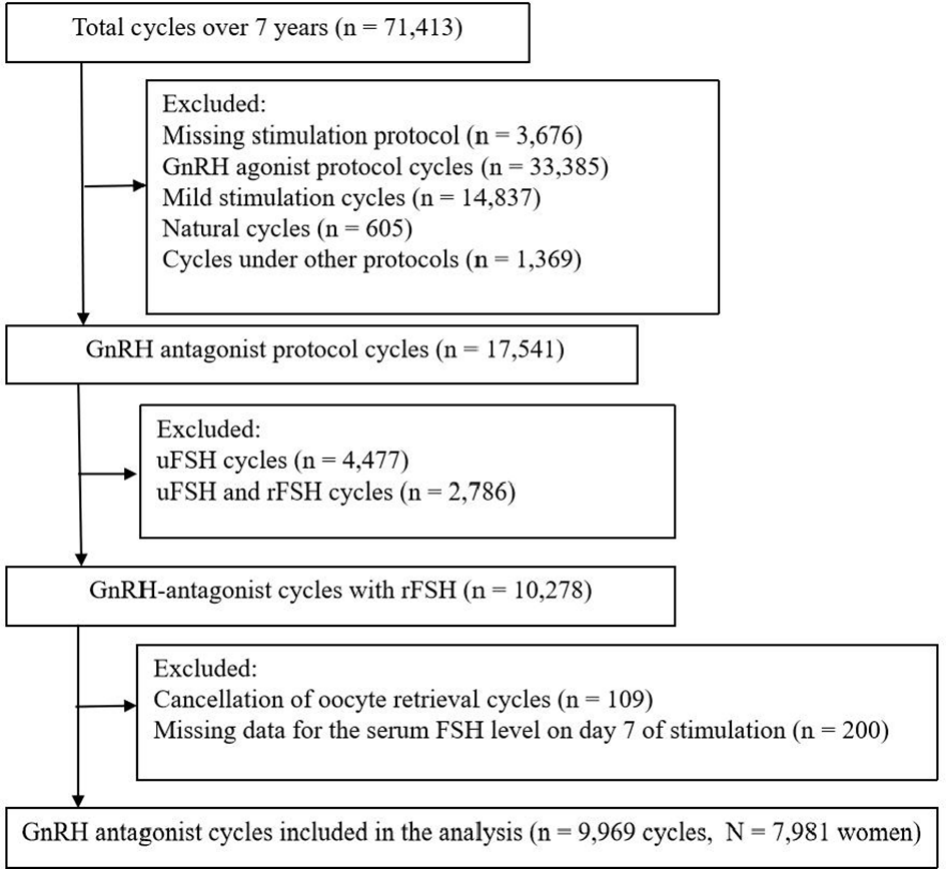
Flow chart for participant selection.

This retrospective study drew on data from patients who underwent fresh IVF or intracytoplasmic sperm injection cycles with gonadotropin-releasing hormone (GnRH) antagonist protocols at Chongqing Health Center for Women and Children, China, between January 2017 and June 2024. Cycles were excluded if they involved urinary gonadotropin administration, lacked serum FSH measurements on Day 7 of stimulation, or were canceled before oocyte retrieval. Data on 9,969 cycles from 7,981 patients were included in the analysis (**Figure 1**).

The definition of polycystic ovary syndrome (PCOS) diagnosis followed the 2003 Rotterdam ESHRE/ASRM guidelines (conference in Rotterdam, Netherlands, March 2003) (17). Poor ovarian reserve (POR) was defined as an anti-Müllerian hormone (AMH) level below 1.2ng/mL or an antral follicle count (AFC) under 5, matching groups 3 and 4 in the POSEIDON classification by Alviggi and colleagues published in 2016 (18).

The Clinical Application and Ethics Committee of Human Assisted Reproductive Technology at Chongqing Health Center for Women and Children checked and approved all study steps (approval number: 2024-RGI06). Given the retrospective design of this study, the requirement for written informed consent was waived by the Ethics Committee.

### 2.2 Ovarian stimulation and assisted reproductive technology procedures

The women in the study received 100–300IU/day recombinant (r)FSH (Puregon, MSD, America; Gonal-f, Merk, Germany; Jinsaiheng, GenSci, China) from day 2 or 3 of the menstruation cycle until the day of administration of human chorionic gonadotropin (hCG). The initial FSH dose was individualized by age, BMI, baseline FSH, AMH, and AFC. Subsequent adjustments were made to reflect hormone profiles and transvaginal ultrasound findings after 4–5 days of stimulation.

Daily administration of 0.25 mg GnRH antagonist (Cetrotide, Merck, Germany; Orgalutran, MSD, USA) was initiated once a dominant follicle reached 12–14 mm, serum estradiol exceeded 600 pg/mL, or luteinizing hormone concentrations were greater than 10 IU/L, and continued until the day that ovulation was triggered (19). When at least three follicles measured ≥ 17 mm, or two follicles reached ≥ 18 mm in diameter, patients received their final antagonist dose in the morning, and ovulation was induced in the evening with 250 ug recombinant hCG (Ovidrel, Serono, Germany). Where more than 19 follicles ≥ 11 mm were observed, ovulation was triggered with 0.2 mg GnRH agonist (Diphereline, Ipsen; Decapeptyl, Ferring, Germany) to minimize the risk of ovarian hyperstimulation syndrome.

Oocyte retrieval was carried out 36–38 hours after ovulation was triggered. It used transvaginal ultrasound-guided aspiration with a single-lumen needle. Intracytoplasmic sperm injection was reserved for couples with severe male factor infertility or a history of failed fertilization.

### 2.3 Hormonal assessment

Blood samples were obtained on cycle day 2 or 3, before the initiation of rFSH and GnRH antagonist treatment, and again on day 7 of ovarian stimulation. All collections were performed between 8:00 a.m. and 11:00 a.m. The interval between the last rFSH injection and blood sampling on day 7 of stimulation was therefore estimated to be approximately 20–25 hours. The serum FSH concentrations were measured through chemiluminescent immunoassay using an Abbott ARCHITECT i2000 chemiluminescence analyzer (Abbott Ireland Diagnostics Division, the Netherlands).

### 2.4 Statistical analysis

Continuous data are shown as mean ± standard deviation (SD), and categorical data as counts and percentages [n (%)]. We divided participants into three groups (low, medium and high) by tertiles of serum FSH on Day 7 of stimulation. Statistical differences between group means and proportions were assessed using one-way ANOVA for normally distributed continuous variables, the Kruskal-Wallis H test for skewed distributions, and Chi-square (χ^2^) tests for categorical variables. Univariate linear regression was used to evaluate the association between serum FSH levels on Day 7 of ovarian stimulation and the number of oocytes retrieved, and identify differences between the three groups. Significant variables were used to adjust subsequent analyses. Both unadjusted and multivariable-adjusted results are reported, with adjustment for woman’s age, duration of infertility, body mass index (BMI), anti-Müllerian hormone (AMH), poor ovarian response (POR), polycystic ovary syndrome (PCOS), type of infertility, baseline FSH (Day1), antral follicle count (AFC), and initial and total gonadotropin dose. Potential non-linear relationships were initially examined using generalized additive models (GAM) with smoothing splines. When the smoothing curve suggested a non-linear association, a two-piece-wise segmented linear regression model (threshold regression model) based on generalized estimating equations (GEE) was used to characterize the threshold effect of Day 7 serum FSH levels. An exchangeable correlation structure was assumed, and robust sandwich estimators were used to account for within−subject correlations. This model has a continuity constraint at the inflection point, and its mean structure is shown as:

When FSH < K: Y = β_0_ + β_1_(FSH − K) + Σγ_i_Z_i_

When FSH ≥ K: Y = β_0_ + β_2_(FSH − K) + Σγ_i_Z_i_

Where K is the inflection point (threshold), β_1_ and β_2_ the slopes of the two segments before and after the threshold, Z_i_ the covariates adjusted in the model, and γ_i_ their corresponding coefficients. This formulation ensures continuity at the inflection point (both segments equal β_0_ when FSH = K).

When the smoothed curve indicated a potential inflection point, a recursive algorithm based on quasi-likelihood maximization was used to automatically identify the optimal threshold. The optimal inflection point was defined as the exposure value that maximized the quasi-likelihood function of the GEE-based two-piece-wise linear regression model. The recursive algorithm contained four sequential steps (1): Global initial screening, with candidate points selected from the 5^th^ to 95^th^ percentiles of the exposure variable at 5% intervals (2); Local range narrowing, where the search range was refined to ± 4 percentiles around the initial optimal candidate, constrained within the 5^th^–95^th^ percentile bounds (3); Fine optimization, where the value yielding the maximum quasi-likelihood was selected from the narrowed candidate set as the optimal inflection point; and (4) Robustness validation, where 1000 iterations of non-parametric bootstrap resampling (sampling with replacement) were performed to estimate the 95% confidence interval (CI) of the inflection point using the percentile method (2.5^th^ and 97.5^th^ percentiles), refitting the GEE-based two-piece-wise model in each bootstrap sample, using the percentile method. Internal validation of the threshold model used 10-fold cross-validation to assess model performance and reduce the risk of overfitting. For GEE models, a quasi-likelihood-based Chi-square test (ANOVA function) was used to compare model fit between the two-piece-wise segmented model and the simple linear model, to evaluate the statistical significance of the threshold effect.

We also carried out sensitivity analyses to check the robustness of our initial findings. First, we used only the first IVF cycle per patient (n = 5,668 cycles). Second, we used only cycles carried out with urinary gonadotropin, rather than recombinant FSH (n = 3,458 cycles), because these alternatives may result in different serum FSH concentrations. All statistical analyses used R software (version 4.2.0; R Foundation for Statistical Computing, Vienna, Austria; https://www.R-project.org) with the mgcv, MASS, geepack, and gdata packages. Threshold effect analysis and customized code generation used EmpowerStats software (X&Y Solutions, Inc., Boston, MA, USA; https://www.empowerstats.com) which supports segmented regression modeling within the GEE framework.

## 3 Results

### 3.1 Participants

The participants had a mean age of 32.74 ± 4.81 years (range: 20–53), mean BMI was 22.34 ± 3.21 kg/m^2^ (range: 13.7–38.4), mean AMH was 3.51 ± 3.81ng/ml (range: 0.01–23.7), mean AFC was 7.99 ± 5.04 (range: 0–46), mean serum FSH level on Day 1 of stimulation was 6.10 ± 1.98 mIU/mL (range: 1.97–15.82), and mean number of oocytes retrieved was 9.40 ± 6.10 (range: 0–57). Overall, 1249 women (12.28%) had PCOS, and 2023 (19.89%) had POR. To mitigate the potential selection bias that may arise from categorizing serum FSH levels with specific values, the three groups were divided using the tertiles of serum FSH levels on Day 7 of the stimulation process (≤ 10.4miu/ml, 10.5–15.4miu/ml, and ≥ 15.5miu/ml). As serum FSH levels on Day 7 of stimulation increased, there was also a gradual increase in patient age, duration of infertility, baseline FSH (Day1), proportion of POR cases, and both initial and cumulative FSH dosages. In contrast, BMI, prevalence of PCOS, AMH levels, and the number of oocytes retrieved showed a decreasing trend (all *P* < 0.001) (**Table 1**).

**Table 1:** Baseline characteristics of participants (n = 9,969 cycles).

Day 7 serum FSH tertile	Low (4.0–10.5miu/ml)	Medium (10.6–15.4miu/ml)	High (≥ 15.5miu/ml)	P-value
Cycles, n	3323	3323	3323	NA
Age of women, years, mean ± SD	29.88 ± 3.92	33.64 ± 4.69	34.64 ± 4.40	< 0.001
Duration of infertility, mean ± SD	4.72 ± 3.23	5.57 ± 4.25	5.64 ± 4.34	< 0.001
BMI, mean ± SD	23.34 ± 3.46	23.07 ± 3.23	21.52 ± 2.59	< 0.001
AMH, mean ± SD	6.75 ± 4.56	2.35 ± 2.26	1.47 ± 1.217	< 0.001
Day 1 serum FSH, mean ± SD	5.10 ± 1.21	6.10 ± 1.71	7.08 ± 2.31	< 0.001
Total FSH dosage, mean ± SD	1523.87 ± 504.25	2133.636 ± 587.37	2490.15 ± 535.43	< 0.001
Initial FSH dosage, mean ± SD	169.91 ± 45.32	247.20 ± 50.35	281.23 ± 32.65	< 0.001
Day 7 serum FSH, mean ± SD	8.1 ± 1.5	13.0 ± 1.4	19.2 ± 3.3	< 0.001
No. of oocytes retrieved, mean ± SD	13.11 ± 7.02	8.71 ± 5.11	6.44 ± 3.62	< 0.001
Infertility factors, n (%)				< 0.001
Tubal disease	2383 (71.7)	2596 (78.1)	2615 (78.7)	
Endometriosis	93 (2.8)	153 (4.6)	199 (6.0)	
Ovulatory disorder	302 (9.1)	63 (1.9)	27 (0.8)	
Male factors	336 (10.1)	289 (8.7)	239 (7.2)	
Unexplained	153 (4.6)	176 (5.3)	193 (5.8)	
Immune infertility	0.0	3 (0.1)	3 (0.1)	
Other	56 (1.7)	43 (1.3)	47 (1.4)	
PCOS, n (%)	1112 (33.5)	132 (4.0)	13 (0.4)	< 0.001
POR, n (%)	112 (3.4)	661 (19.9)	1226 (36.9)	< 0.001

Data shown as mean ± SD or n (%), unless otherwise stated.

BMI, body mass index; AMH, anti-Müllerian hormone; AFC, antral follicle counts; PCOS, polycystic ovary syndrome; POR, poor ovarian reserve; Day 1 serum FSH, serum FSH on Day 1 of stimulation; Day 7 serum FSH, serum FSH on Day 7 of stimulation.

### 3.2 Univariate analysis for number of oocytes retrieved

The univariate analysis results are shown in **Table 2**. The women’s age, BMI, duration of infertility, serum FSH level on both Day 1 and Day 7 of stimulation, both initial and total FSH dosage, endometriosis, and POR were all negatively correlated with the number of oocytes retrieved. AMH, PCOS, male factor infertility and ovulatory disorder were positively correlated with the number of oocytes retrieved.

**Table 2:** Univariate analysis for number of oocytes retrieved (n = 9,969 cycles).

Exposure	Mean ± SD or N (%)	Effect size (β)	*P* value
Woman’s age, years	32.74 ± 4.81	−0.39 (−0.41, −0.36)	< 0.0001
Duration of infertility	5.32 ± 4.00	−0.15 (−0.18, −0.12)	< 0.0001
Woman’s BMI	22.65 ± 3.21	−0.07 (−0.11, −0.03)	0.0005
AMH	3.51 ± 3.81	0.91 (0.89, 0.94)	< 0.0001
Day 1 serum FSH	6.20 ± 1.98	−1.13 (−1.19, −1.07)	< 0.0001
AFC	7.99 ± 5.04	0.71 (0.69, 0.73)	< 0.001
Infertility factors			
Tubal disease	7594 (76.2%)	*Ref*	
Endometriosis	445 (4.5%)	−1.3 (−1.9, −0.7)	< 0.0001
Ovulatory disorder	392 (3.9%)	3.8 (3.2, 4.4)	< 0.0001
Male factors	864 (8.7%)	0.9 (0.5, 1.3)	< 0.0001
Unexplained	522 (5.2%)	−0.4 (−0.9, 0.1)	0.1300
Immune infertility	6 (0.1%)	0.3 (−3.9, 4.5)	0.9008
Other	142 (1.4%)	1.7 (0.7, 2.6)	0.0010
POR	1999 (20.1%)	−4.9 (−5.2, −4.6)	< 0.0001
PCOS	1257 (12.6%)	5.2 (4.8, 5.5)	< 0.0001
Total FSH dosage	2053.5 ± 677.4	−0.004 (−0.004, −0.003)	< 0.0001
Initial FSH dosage	232.9 ± 63.6	−0.051 (−0.053, −0.050)	< 0.0001

BMI, body mass index; AMH, anti-Müllerian hormone; PCOS, polycystic ovary syndrome; POR, poor ovarian reserve.

### 3.3 Association between serum FSH levels on day 7 of stimulation and number of oocytes retrieved

We used multivariable linear regression models to assess the relationship between the serum FSH levels on Day 7 of stimulation and the number of oocytes retrieved. The unadjusted and adjusted models are shown in **Table 3**. In the crude model, serum FSH levels on Day 7 of stimulation were negatively associated with the number of oocytes retrieved (β = −0.531, 95% confidence interval [CI]: −0.552, −0.510, *P* < 0.0001). In the model adjusted for woman’s age, duration of infertility, BMI, baseline FSH (Day1), AMH, POR, PCOS, infertility factors, AFC, initial and total FSH dose, the results remained consistent (β = –0.047, 95% CI: –0.078, −0.017, *P* = 0.0025).

**Table 3:** Relationship between day 7 serum FSH and number of oocytes retrieved in different models (n = 8,042 cycles).

Outcome	Crude model		Adjusted model	
	β (95% CI)	*P-value*	β (95% CI)	*P-value*
Day 7 serum FSH	−0.53 (−0.55, −0.51)	< 0.0001	−0.05 (−0.08, −0.02)	0.0025
Day 7 serum FSH level (quantiles) (mIU/mL)				
Low	*Ref*		*Ref*	
Middle	−4.37 (−4.63, −4.11)	< 0.0001	0.14 (−0.19, 0.47)	0.3948
High	−6.64 (−6.90, −6.38)	< 0.0001	−0.66 (−1.03, −0.28)	0.0006
*P* for trend	< 0.001			

CI, confidence interval; Ref, reference.

The model was adjusted for woman’s age, duration of infertility, body mass index, anti-Müllerian hormone, serum FSH level on Day 1 of stimulation, polycystic ovary syndrome, poor ovarian reserve, infertility factors, and initial and total FSH dosage.

### 3.4 Non-linear relationship between serum FSH levels on day 7 of stimulation and number of oocytes retrieved

**Figure 2:**
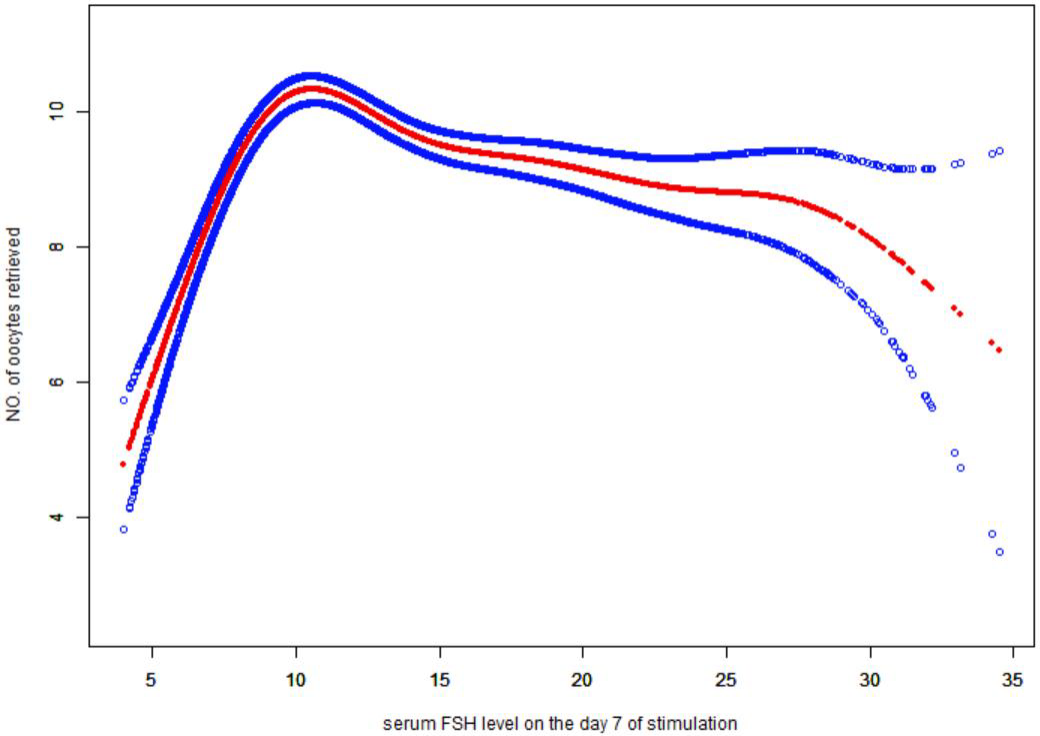
Association between serum FSH level on Day 7 of stimulation (mIU/mL) and number of oocytes retrieved (n =8,042 cycles). A threshold, non-linear association was found between serum FSH level on Day 7 of stimulation and number of oocytes retrieved (p < 0.0001) using a generalized additive model (GAM). The solid red line represents the smooth curve fit between variables. The blue bands show the 95% confidence interval of the fit. All were adjusted for woman’s age, duration of infertility, body mass index, anti-Müllerian hormone, serum FSH level on Day 1 of stimulation, polycystic ovary syndrome, poor ovarian reserve, infertility factors, AFC, and initial and total FSH dosage.

Serum FSH levels on Day 7 of stimulation were considered a continuous variable, and we therefore assessed a potential non-linear association with the number of oocytes retrieved. After adjustment woman’s age, duration of infertility, BMI, baseline FSH (Day 1), AMH, POR, PCOS, infertility factors, AFC, initial and total gonadotropin dose, we saw a threshold non-linear relationship (**Figure 2**) (p < 0.0001) in a generalized additive model (GAM). The solid red line shows the smooth curve fit between variables. The blue bands show the 95% confidence interval of the fit, all woman’s age, duration of infertility, BMI, baseline FSH (Day 1), AMH, POR, PCOS, infertility factors, AFC, initial and total gonadotropin dose. Using a two-piece-wise linear regression model based on GEE, the inflection point was estimated at 9.13 mIU/mL. To the right of the inflection point, the number of oocytes retrieved decreased by 0.07 per 1 mIU/mL increase in Day 7 serum FSH levels (effect size β = −0.07, 95% CI −0.10 to −0.05 and P < 0.0001). To the left of the inflection point, the number of oocytes retrieved increased by 1.18 per 1 mIU/mL increase in Day 7 serum FSH levels (effect size β = 1.18, 95% CI: 0.95 to 1.41 and P < 0.0001) when adjusted for woman’s age, duration of infertility, BMI, baseline FSH (Day 1), AMH, POR, PCOS, infertility factors, AFC, initial and total gonadotropin dose (**Table 4**). The 10-fold cross-validation confirmed the robustness of the two-piece-wise segmented linear regression model (**Supplementary Figure 1**).

**Table 4:** Threshold effect analysis of relationship between day 7 serum FSH level and number of oocytes retrieved using piece-wise linear regression in cycles using recombinant FSH (n = 8,042 cycles). .

Day 7 serum FSH	Effect size (β)	95% CI	*P* value
Breakpoint K	9.13	7.99, 9.75*	
< 9.13mIU/mL	1.18	0.95, 1.41	< 0.0001
≥ 9.13mIU/mL	−0.07	−0.10, −0.05	< 0.0001
Logarithmic likelihood ratio test			< 0.001

*95% CI for the breakpoint: 1000 bootstrap resamples.

Adjusted for woman’s age, duration of infertility, body mass index, anti-Müllerian hormone, serum FSH level on Day 1 of stimulation, polycystic ovary syndrome, poor ovarian reserve, infertility factors, AFC, and initial and total FSH dosage.

Effect: the number of oocytes retrieved.

Cause: serum FSH level on Day 7 of stimulation.

CI, confidence interval.

### 3.5 Subgroup analyses

Subgroup analyses showed significant effect modifications by PCOS, AMH, BMI, and woman’s age on the association between serum FSH level on Day 7 of stimulation and number of oocytes retrieved (all P for interaction < 0.05). In contrast, no significant interaction was observed for POR (P for interaction = 0.37) (**Table 5**). There was an inverse association in women without PCOS (β = −0.04, 95% CI −0.08 to −0.01), and a pronounced positive association in women with PCOS (β = 1.01, 95% CI 0.85 to 1.17) (P for interaction < 0.001). For AMH, the association shifted from negative in the lowest stratum (< 1.2 ng/mL: β = −0.08, 95% CI −0.13 to −0.02) to positive in the highest stratum (≥ 5 ng/mL: β = 0.58, 95% CI 0.46 to 0.70), with a highly significant interaction (P for interaction < 0.001). For BMI, there was a negative association in underweight women (< 18.5 kg/m^2^: β = −0.14, 95% CI −0.27 to −0.01), but no significant association in women with BMI ≥ 18.5 kg/m^2^ (P for interaction = 0.046). There was no significant association among women younger than 35 years, but a marginally significant inverse association in women aged 35 years or older (P for interaction = 0.03). These findings were visually corroborated by stratified smooth curves constructed using generalized additive models (**Supplementary Figure 2–6**). Consistent with the non-significant interaction, smooth curves stratified by POR overlapped substantially.

**Table 5:** Subgroup analysis of the association between serum FSH level on day 7 of stimulation and number of oocytes retrieved (n = 8,042 cycles).

Subgroup	β (95% CI)	*P* value	*P* interaction
Age (years)			0.030
< 35, n = 5,307	0.03 (−0.02, 0.08)	0.279	
≥ 35, n = 2,735	−0.05 (−0.11, 0)	0.054	
AMH (ng/mL)			< 0.001
< 1.2, n = 2,505	−0.08 (−0.13, −0.02)	0.004	
1.2–4.9, n = 3,633	−0.02 (−0.07, 0.04)	0.528	
≥ 5, n = 1,904	0.58 (0.46, 0.70)	< 0.001	
BMI (kg/m²)			0.046
< 18.5, n = 604	−0.14 (−0.27, −0.01)	0.03	
≥ 18.5, n = 7,438	−0.01 (−0.04, 0.03)	0.74	
POR			0.373
No, n = 6,397	−0.01 (−0.05, 0.04)	0.792	
Yes, n = 1,645	−0.04 (−0.11, 0.02)	0.211	
PCOS			< 0.001
No, n = 7,008	−0.04 (−0.08, −0.01)	0.028	
Yes, n = 1,034	1.01 (0.85, 1.17)	< 0.001	

Adjusted for woman’s age, duration of infertility, body mass index, anti-Müllerian hormone, serum FSH level on Day 1 of stimulation, polycystic ovary syndrome, poor ovarian reserve, infertility factors, and initial and total FSH dosage. In analyses stratified by poor ovarian reserve or polycystic ovary syndrome, the stratifying variable was excluded from adjustment.

AMH, anti-Müllerian hormone; BMI, body mass index; POR, poor ovarian reserve; PCOS, polycystic ovary syndrome.

### 3.6 Sensitivity analyses

**Figure 3:**
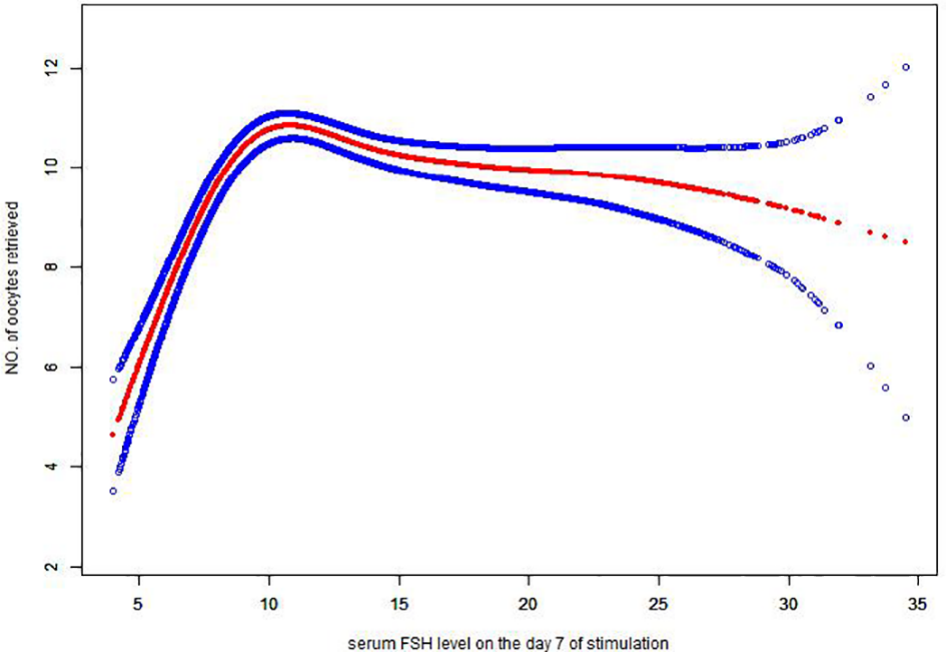
Association between serum FSH level on Day 7 of stimulation (mIU/mL) and number of oocytes retrieved in GnRH antagonist protocols using only the first cycle per patient (n = 5,668 cycles). There was a threshold, non-linear association between serum FSH level on Day 7 of stimulation and number of oocytes retrieved (p < 0.0001) in a generalized additive model (GAM). The solid red line represents the smooth curve fit between variables. The blue bands represent the 95% confidence interval of the fit. All were adjusted for woman’s age, duration of infertility, body mass index, anti-Müllerian hormone, serum FSH level on Day 1 of stimulation, polycystic ovary syndrome, poor ovarian reserve, infertility factors, AFC, and initial and total FSH dosage.

In the sensitivity analysis using only the first cycle per patient, the relationship between serum FSH levels on Day 7 of stimulation and the number of oocytes retrieved remained non-linear (**Figure 3**), and the inflection point was estimated at 9.19miu/ml (**Table 6**).

**Table 6:** Threshold effect analysis of serum FSH level on day 7 of stimulation and number of oocytes retrieved using piece-wise linear regression on first cycles only (n = 5,668 cycles).

Day 7 serum FSH	Effect size (β)	95% CI	*P* value
Breakpoint K	9.19	7.98, 9.79*	
< 9.19mIU/mL	1.15	0.98, 1.33	< 0.0001
≥ 9.19mIU/mL	−0.08	−0.12, −0.03	< 0.0001
Logarithmic likelihood ratio test			< 0.001

*95% CI for the breakpoint: 1000 bootstrap resamples; CI, confidence interval.

Adjusted for woman’s age, duration of infertility, body mass index, anti-Müllerian hormone, serum FSH level on Day 1 of stimulation, polycystic ovary syndrome, poor ovarian reserve, infertility factors, and initial and total FSH dosage.

Effect: the number of oocytes retrieved.

Cause: serum FSH level on Day 7 of stimulation.

The analysis using only cycles involving urinary gonadotropins, not recombinant FSH, included 3,458 cycles. The relationship between serum FSH level on Day 7 of stimulation and the number of oocytes retrieved was consistent with the main analysis, and showed a non-linear threshold effect (**Supplementary Figure 7**). Using a two-piece-wise linear regression model, the inflection point (K) was identified as 10.45 mIU/mL. When serum FSH level on Day 7 of stimulation was < 10.45 mIU/mL, each 1 mIU/mL increase was associated with an increase in the number of oocytes retrieved (β = 1.38, 95% CI: 1.14 to 1.62, P < 0.001). However, when serum FSH level on Day 7 of stimulation was ≥ 10.45 mIU/mL, the effect was not statistically significant (β = 0.002, 95% CI: −0.048 to 0.045, P = 0.942) (**Supplementary Table 1**). The log-likelihood ratio test gave P < 0.001, suggesting that the two-piece-wise model was superior to the linear model. To further validate the stability of the inflection point, we performed 1,000 bootstrap resamples, and the results showed a 95% confidence interval of 10.17–12.03 mIU/mL for the inflection point.

## 4 Discussion

This study, based on a large sample size dataset, systematically and rigorously investigated the relationship between serum FSH levels on Day 7 of ovarian stimulation and the number of oocytes retrieved in GnRH antagonist protocols using recombinant FSH. We found a significant non-linear relationship between these two variables. Below a threshold, Day 7 serum FSH was positively correlated with the number of oocytes retrieved, and above the threshold, the correlation became negative. The estimated threshold is 9.13 mIU/mL. This suggests the existence of an optimal serum FSH range during controlled ovarian stimulation that elicits the best ovarian response.

We found a negative correlation between serum FSH levels and the number of oocytes retrieved, which remained consistent in the adjusted model (β = −0.047, 95% CI −0.078 to −0.017, P = 0.0025). However, two-piece-wise linear regression analysis revealed a strong bidirectional correlation between serum FSH levels and the number of oocytes retrieved. Below the threshold Day 7 serum FSH level of 9.13 mIU/mL, β = 1.18, P < 0.0001, and above the threshold, β = −0.07, P < 0.0001. This makes clear that applying two-piece-wise model analysis in the context of a non-linear relationship is essential to clearly explain the association between the two variables. We also used 1,000 bootstrap resamples to calculate the confidence interval for the inflection point and added 10-fold cross-validation, both of which supported the stability and reliability of the threshold. Stratified analysis showed significant effect modification by PCOS, AMH, BMI, and age on the association.

Previous studies have reported conflicting findings about the relationship between serum FSH levels and the number of oocytes retrieved. Some studies (1, 20) have indicated that higher doses of FSH lead to higher serum FSH concentrations and increased oocyte yield. One estimated that each 50 IU increase in daily FSH dose increased the number of oocytes retrieved by approximately one (21). However, other studies showed different results. One using multivariate analysis found a significant negative correlation between oocyte yield and serum FSH levels (4). Another reported that in ovarian stimulation cycles using a fixed daily dose of 150 IU rFSH, poor ovarian response was not correlated with serum FSH levels on the day of hCG administration (8). However, that study also found that in high-responder patients, reducing the FSH dose led to a corresponding decrease in serum FSH levels and lower ovarian response.

These findings are inconsistent and even contradictory. However, they may be explained by our finding of a significant non-linear association. This suggests the existence of an optimal threshold level, beyond which elevated FSH levels are no longer associated with improved oocyte yield. We suggest that previous studies may have failed to consider the bidirectional regulatory nature of the relationship between serum FSH levels and oocyte yield. During ovarian stimulation, serum FSH levels are closely related to the administered dose of exogenous gonadotropins. Previous studies on the relationship between FSH dose and ovarian response have also identified an optimal threshold for exogenous gonadotropin dosage: below this threshold, increasing the gonadotropin dose enhances oocyte yield, but once this threshold is exceeded, increasing the dose further not only fails to increase oocyte yield but may even negatively impact oocyte quality and outcomes (6, 7, 9).

One interesting aspect of our findings is the relatively low level of the threshold. Our sample was initially divided into three groups (low, medium and high) using tertiles of serum FSH levels on Day 7 of stimulation. The threshold value of 9.13 mIU/mL falls into the lowest tertile, which ranged from 4.0 to 10.5 mIU/mL. The three groups had no significance for outcomes in the study, and were only used to identify potential adjustment variables. However, the location of the threshold in the lowest tertile means that more than two-thirds of the women in this study had Day 7 serum FSH levels above the threshold. If this finding is confirmed by prospective studies, it could suggest that excess FSH may be more important than insufficient levels in determining the success of oocyte retrieval in fertility treatment.

*In vitro* studies culturing animal preantral and antral follicles have found that low concentrations of FSH significantly increase FSH receptor expression, and high concentrations lead to receptor down-regulation (22). These factors may help to explain our findings. Once FSH levels reach a certain threshold, further elevation may inhibit FSH receptor expression, affecting the number of growing follicles and leading to a reduction in oocyte yield. However, other studies have indicated that the density and regulation of FSH and luteinizing hormone receptors on granulosa cells are associated with women’s age, and their mechanisms operate independently *in vivo*, with a relatively weak correlation with FSH dosage (23). We therefore cannot rule out the possibility that the low oocyte yield may be due to reduced receptor numbers in granulosa cells and poor follicular development in older women or those with lower ovarian reserve. Alternatively, it may result from low FSH receptor expression causing slower FSH clearance and higher FSH levels. Prospective studies are urgently needed to verify whether maintaining serum FSH within an optimal range through exogenous dose adjustments can improve ovarian response, embryo developmental potential, and assisted reproductive outcomes.

This study has some limitations. Its retrospective design can only reveal associations between variables. No causal inferences can therefore be drawn, and prospective studies are needed to verify whether our findings may be clinically applied. Additionally, it was a single-center study, the study population showed strong homogeneity, and only antagonist protocols were included, which may have introduced selection bias. Confounding factors may also have interfered with the observed associations. In particular, we cannot rule out confounding by indication, because higher doses are often given to women expected to have a poor response to FSH. The study also only included cycles using recombinant FSH, which may introduce potential selection bias. However, we conducted a sensitivity analysis on cycles using urinary FSH, and found that the association between serum FSH levels on Day 7 of stimulation and oocyte yield was consistent, verifying the stability of the core conclusion. The dataset in this study contained repeated cycle data, which could potentially introduce bias, but we carried out a generalized estimating equation (GEE) analysis, and the results were again consistent with the piece-wise regression, suggesting that the conclusions are robust and reliable. Similarly, 10-fold cross-validation was used as a robust internal validation strategy to assess model robustness and mitigate the risk of overfitting. We also carried out a sensitivity analysis using data only from the first oocyte retrieval cycle, and the conclusions remained consistent. There were also some issues with the data collection. For example, the exact timing of blood sample collection was not recorded. Based on the workflow, it was estimated to be 20–25 hours after the last FSH injection for serum FSH level measurement, but this timing will have varied. We also could not obtain information about mature oocyte count, fertilization rate, usable embryo rate, or blastocyst yield because of time limitations. Future studies should consider gathering this information to provide more useful information about likely success of IVF. Finally, testing errors during the serum FSH assay process may have contributed to deviations in the determined optimal reference range for FSH levels, and our results need clinical verification in prospective studies.

## 5 Conclusion

We found that in GnRH antagonist protocols, the number of oocytes retrieved showed a significant non-linear relationship with serum FSH levels on Day 7 of ovarian stimulation: when serum FSH was relatively low, the oocyte yield increased significantly with rising FSH levels. However, after reaching a certain level, the oocyte yield decreased even when FSH levels continued to rise (P < 0.0001). The threshold for optimal serum FSH identified was 9.13 mIU/mL. However, factors such as the type of exogenous medication used, injection timing, FSH assay methods, and testing errors may all influence measured results, and this threshold should be regarded as an initial reference value or hypothesis, requiring prospective studies for verification. Our finding suggests that it may in future be possible to adjust medication doses based on an optimal FSH level, as a way to improve ovarian response and assisted reproductive outcomes. However, this hypothesis requires confirmation through prospective studies. Future research should also explore the molecular mechanisms that may underlie this threshold effect.

## Data Availability

The original contributions presented in the study are included in the article/**Supplementary Material**. Further inquiries can be directed to the corresponding author.
