# Supplementary figures and images for: High serum FSH levels on day 7 of stimulation are negatively associated with oocyte retrieval in GnRH antagonist protocols: real-world evidence from 9,969 IVF cycles in China
Source: Front Endocrinol (Lausanne). 2026 May 5;17:1757107. doi: 10.3389/fendo.2026.1757107 (PMC13183537; doi:10.3389/fendo.2026.1757107)

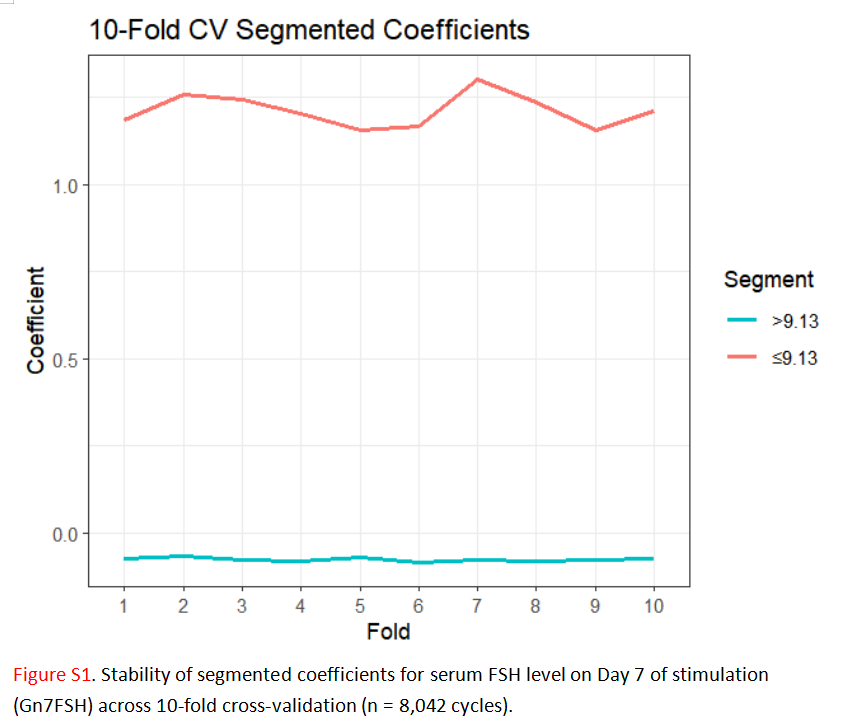

Supplement: Supplementary file 1 [file Image1.jpeg]

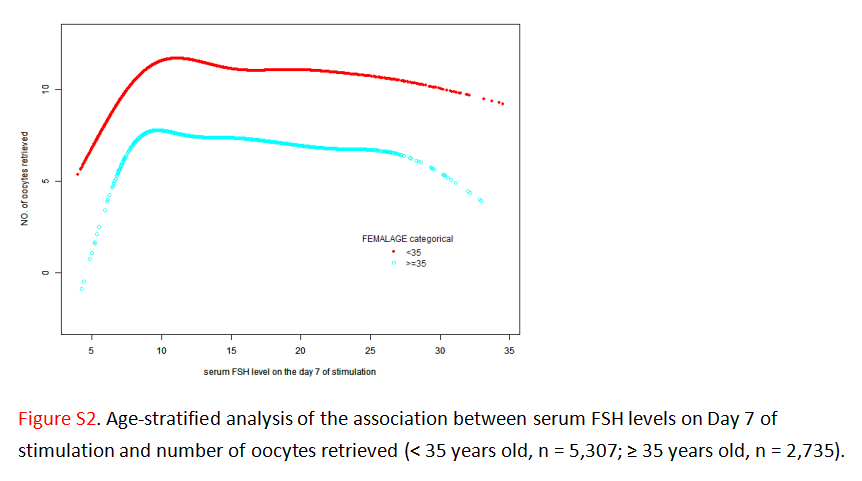

Supplement: Supplementary file 2 [file Image2.jpeg]

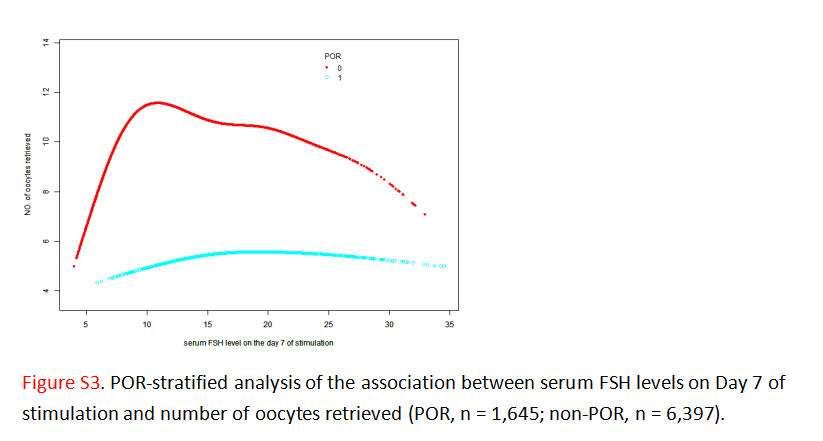

Supplement: Supplementary file 3 [file Image3.jpeg]

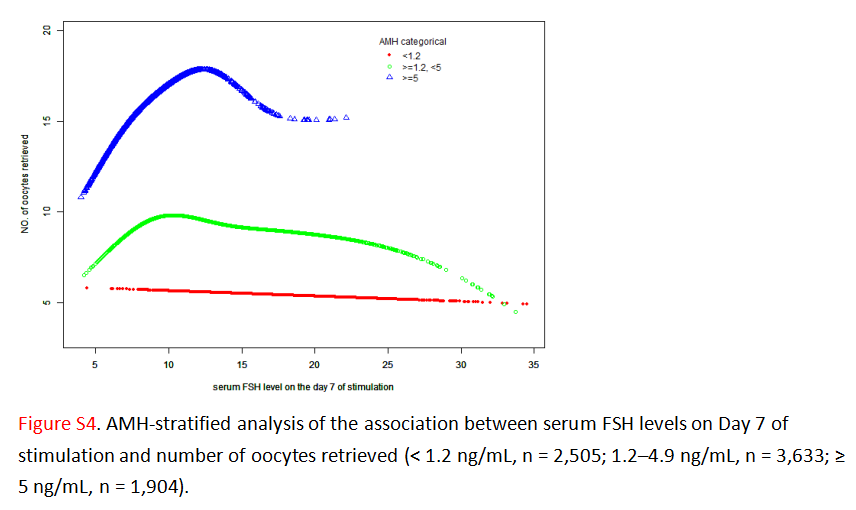

Supplement: Supplementary file 4 [file Image4.jpeg]

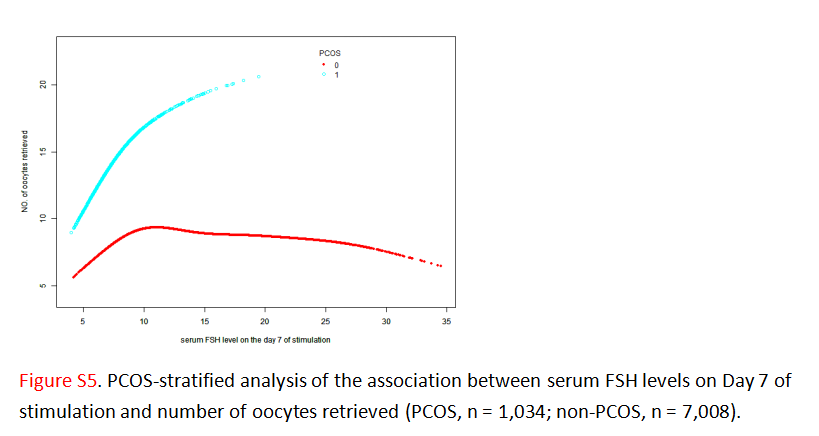

Supplement: Supplementary file 5 [file Image5.jpeg]

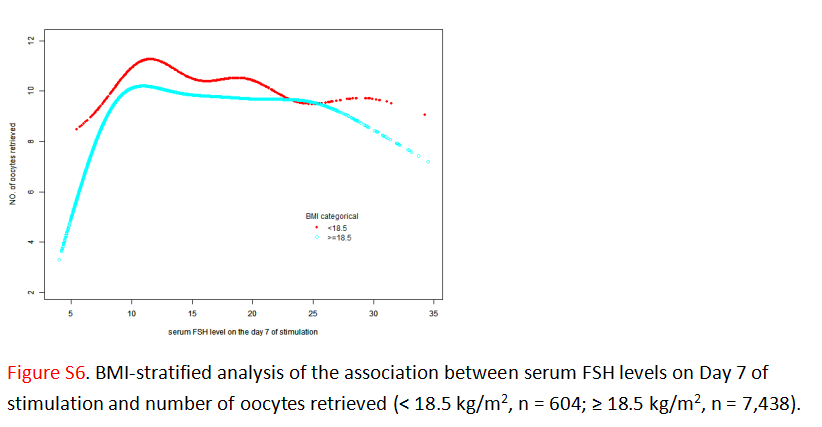

Supplement: Supplementary file 6 [file Image6.jpeg]

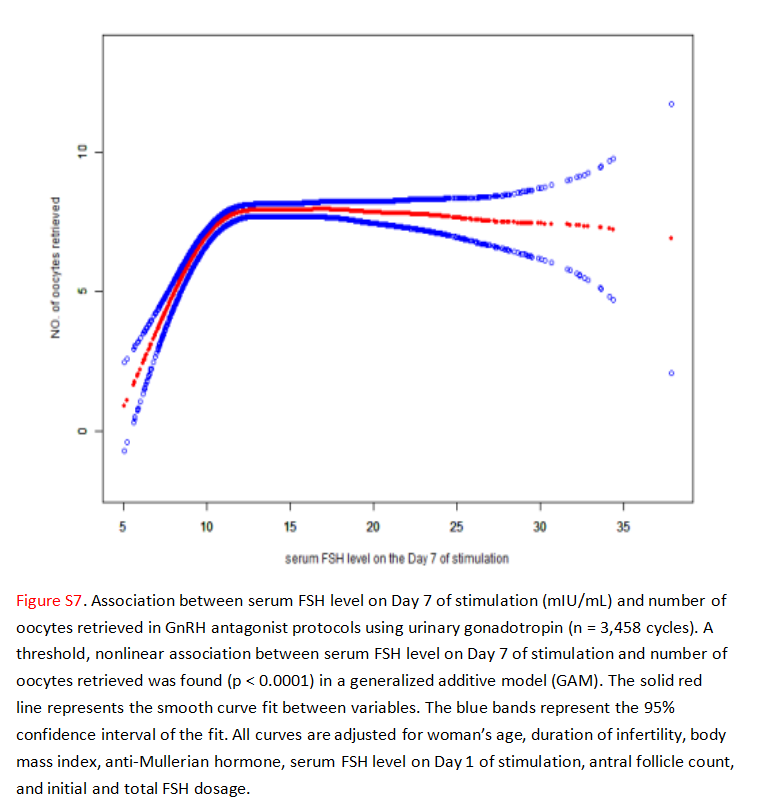

Supplement: Supplementary file 7 [file Image7.jpeg]

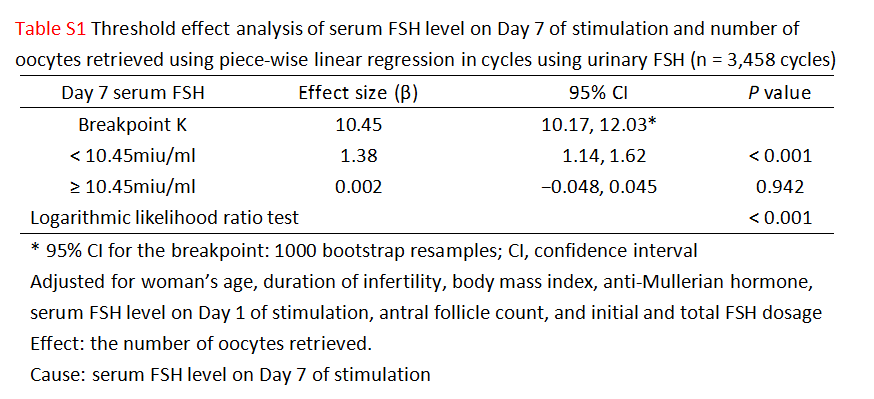

Supplement: Supplementary file 8 [file Image8.jpeg]
